# Functional Segregation of the Middle Temporal Visual Motion Area Revealed With Coactivation-Based Parcellation

**DOI:** 10.3389/fnins.2020.00427

**Published:** 2020-05-27

**Authors:** Jingjing Gao, Min Zeng, Xin Dai, Xun Yang, Haibo Yu, Kai Chen, Qingmao Hu, Jinping Xu, Bochao Cheng, Jiaojian Wang

**Affiliations:** ^1^School of Information and Communication Engineering, University of Electronic Science and Technology of China, Chengdu, China; ^2^Department of Radiology, Pidu District People’s Hospital, Chengdu, China; ^3^School of Automation, Chongqing University, Chongqing, China; ^4^School of Public Affairs, Chongqing University, Chongqing, China; ^5^Department of Acupuncture and Moxibustion, Shenzhen Traditional Chinese Medicine Hospital, Shenzhen, China; ^6^School of Automation Engineering, University of Electronic Science and Technology of China, Chengdu, China; ^7^Institute of Biomedical and Health Engineering, Shenzhen Institutes of Advanced Technology, Chinese Academy of Sciences, Shenzhen, China; ^8^CAS Key Laboratory of Human-Machine Intelligence-Synergy Systems, Shenzhen Institutes of Advanced Technology, Chinese Academy of Sciences, Shenzhen, China; ^9^Department of Radiology, West China Second University Hospital of Sichuan University, Chengdu, China; ^10^School of Life Sciences and Technology, University of Electronic Science and Technology of China, Chengdu, China; ^11^Center for Language and Brain, Shenzhen Institute of Neuroscience, Shenzhen, China

**Keywords:** MT, visual motion perception, meta-analysis, coactivation-based parcellation, meta-analytic connectivity mapping

## Abstract

Traditionally, the visual motion area (MT) is considered as a brain region specialized for visual motion perception. However, accumulating evidence showed that MT is also related to various functions, suggesting that it is a complex functional area and different functional subregions might exist in this area. To delineate functional subregions of this area, left and right masks of MT were defined using meta-analysis in the BrainMap database, and coactivation-based parcellation was then performed on these two masks. Two dorsal subregions (Cl1 and Cl2) and one ventral subregion (Cl3) of left MT, as well as two dorsal-anterior subregions (Cl1 and Cl2), one ventral-anterior subregion (Cl3), and an additional posterior subregion (Cl4) of right MT were identified. In addition to vision motion, distinct and specific functions were identified in different subregions characterized by task-dependent functional connectivity mapping and forward/reverse inference on associated functions. These results not only were in accordance with the previous findings of a hemispheric asymmetry of MT, but also strongly confirmed the existence of subregions in this region with distinct and specific functions. Furthermore, our results extend the special role of visual motion perception on this area and might facilitate future cognitive study.

## Introduction

The human being acquires outer information mainly relying on visual inputs. Visual motion perception is primarily modulated by the visual motion area (MT), which is a brain region specialized for the perception of motion in the visual modality. The first discovery of MT comes from the study of stimulating sensitive area of visual movement in the monkey brain (Allman and Kaas, 1971; Dubner and Zeki, 1971; Zeki, 1974). Subsequently, a positron emission tomography (PET) study confirmed the existence of a homologous counterpart in human brain (Zeki et al., 1991). Although the MT is identified in human brain, the exact location of MT is still under debate. Using PET technology, Zhang et al. (2007) studied 12 normal movement and static visual tasks and consistently found that the MT area was located in the occipital lobe in spite of individual variability. This area was exactly located in the junction between the ascending limb of the inferior temporal sulcus and the lateral occipital sulcus (Watson et al., 1993). Using functional magnetic resonance imaging (fMRI), Dumoulin et al. (2000) found that the location of MT mainly included three sulcus: the inferior temporal sulcus (11%), temporal sulcus rising branch (53%), and the posterior continuations of inferior temporal sulcus (46%). MT was recently further delineated by analysis of myelination, and MT has significantly different myelination compared to surrounding tissues (Annese et al., 2004).

The traditional view of MT is that this area mainly responds to visual motion. A large number of recent studies have demonstrated that this area is also related to motion of auditory and tactile (Howard et al., 1995; Poirier et al., 2006; Ricciardi et al., 2007; Watkins et al., 2013; Abdollahi et al., 2014). Different ways of stimulation result in different response areas of MT (Morrone et al., 2000). Using fMRI, Smith et al. (2006) further demonstrated that there are different functional subregions in MT: the lateral MT was significantly activated by the optic flow stimuli from the contralateral side, whereas the other subregion, medially MST, was significant activated by the optic flow stimuli from the same side (Smith et al., 2006). Moreover, MT was reported to be involved into spatial deep perception, shape detection, and binocular rivalry (Ferri et al., 2013). All these evidence suggested that MT is a complex functional area and different functional subregions exist in this area.

More and more studies have demonstrated that the brain functions were determined by its different connectivity patterns with other brain areas (Wang et al., 2012, 2015a; Xu et al., 2015, 2019a). Using anatomical and resting-state functional connectivity-based parcellation approach, many brain areas have been subdivided into different functional subregions (Wang et al., 2015b, 2016; Xu et al., 2019b). Recently, Eickhoff et al. (2011) proposed task-related coactivation-based parcellation approach to parcellate the brain with BrainMap database^1^ to characterize the functional organization of the brain under task. Coactivation-based parcellation results showed similarities with the findings derived from anatomical and resting-state functional connectivity-based parcellation (Wang et al., 2015b, 2017, 2019). Moreover, given coactivation-based parcellation characterizing the task-related connectivity, it is thus better to investigate the brain functional areas that do not have clear anatomical boundary.

In our study, we first defined left and right functional masks of MT using meta-analysis in the BrainMap database. Then, we performed coactivation-based parcellation of left and right MT to identify functional subregions. Finally, we characterized the task-related connectivity and functions for each subregion using meta-analytic connectivity mapping (MACM).

## Materials and Methods

### Definition of MT Area Masks

Motion area is specific for visual motion and approximately located in the junction of the posterior middle temporal gyrus (MTG) and occipital gyrus (Smith et al., 2006). Given no consensus of neuroanatomical landmarks to define the location of MT, thus, we used meta-analysis of visual motion task in BrainMap database to identify MT. After obtaining the coordinates of the experiments for visual motion, activation likelihood estimation (ALE) was applied to model the functional activation, and *p* < 0.001 with false discovery rate (FDR) correction was used to identify the functional activations to define the functional masks for MT for parcellation.

### Coactivation-Based Parcellation

Coactivation-based parcellation approach was used to identify the functional subregions of MT in this study. The whole-brain coactivation connectivity calculated using MACM was further used to define the coactivation profile for each MT voxel with BrainMap database (Laird et al., 2009, 2011). Only PET and fMRI experiments reporting stereotaxic coordinates in healthy subjects were entered into our analyses. In BrainMap database, the tasks ranged from executive functions to sensorimotor processing and cognition, such as inhibition control, working memory, and language processing. Voxels in the neighborhood of each seed voxel in MT were pooled and used to define the task-related coactivation pattern. By computing and sorting the Euclidian distances between a given seed voxel and any reported activation coordinate, those experiments reporting activation coordinates closest to the present seed voxel were identified with the extent of the spatial filter from 20 to 200 experiments in steps of 5. Next, an ALE meta-analysis of the experiments associated with that particular voxel was performed, and the ALE scores were taken as the whole-brain coactivation pattern (Eickhoff et al., 2009, 2012; Turkeltaub et al., 2012; Bzdok et al., 2013; Cieslik et al., 2013). The coactivation patterns for all the seed voxels were combined into an *N* × *M* matrix, where *N* is the number of seed voxels in the MT and *M* is the number of whole-brain voxels. The similarity was defined using one minus the correlation between each pair of the coactivation patterns of the seed voxels in MT. Finally, MT parcellation was performed using K-means clustering method with *K* = 2, 3, …, 7 in the optimal filter (Clos et al., 2013). The optimal filter range was determined by assessing the consistency of the cluster assignments for individual voxels across different filter sizes, and the range with the lowest number of deviants was selected as the optimize.

### Determination of the Number of Clusters

The index of hierarchically inconsistent voxels was used to determine the optimal number of clusters of the MT. The index of hierarchically inconsistent voxels quantifies the percentage of voxels not related to the dominant parent cluster compared to the *K*-1 clustering number. The lowest lost voxel was considered as the optimal clustering solution (Kahnt et al., 2012; Clos et al., 2013).

### Whole-Brain Coactivation Patterns of Each Subregion

Structure-based meta-analysis was used to map the task-based coactivation patterns for each subregion of MT in the BrainMap database. To map the coactivation pattern, at least one focus of activation in a particular subregion was pooled (Eickhoff et al., 2010; Robinson et al., 2010; Laird et al., 2013). An ALE meta-analysis on the experiments and statistical inference were performed to identify brain regions that significantly coactivated with a particular subregion. Then, the ALE score was compared to a null-distribution to determine the above-chance convergence between experiments (Eickhoff et al., 2009). The ALE scores activated within a particular subregion were tested against the ALE scores obtained under this null-distribution, yielding a *p*-value on the basis of the proportion of equal or higher random values (Eickhoff et al., 2012). These non-parametric *p*-values were finally converted to *z*-scores and thresholded at *p* < 0.05 (cluster-level FWE-corrected, cluster-forming threshold at voxel-level, *p* < 0.001).

### Specific Coactivation Pattern for Each Subregion

The specific coactivation pattern was calculated to identify the unique task-related coactivation patterns for each MT subregion compared to other subregions. The specific coactivation patterns were the brain areas that were significantly more coupled with a given subregion than that with any of the others.

### Functional Characterization of Each Subregion

Each MT subregion was functionally characterized based on behavioral domain and paradigm class using the BrainMap database. Functional characterization of each MT subregion was determined using forward and reverse inferences (Bzdok et al., 2013; Cieslik et al., 2013; Clos et al., 2013; Rottschy et al., 2013; Wang et al., 2015b). In the forward inference approach, the functional profile of a specific subregion was determined by identifying the domains or subdomains for which the probability of activation was significantly higher than the overall chance of activation in that particular subregion. Significance was established using a binomial test (*p* < 0.05 corrected for multiple comparisons using FDR method) (Eickhoff et al., 2011). In the reverse inference approach, the functional profile of a subregion was determined by identifying the most likely behavioral domains and paradigm classes associated with activation in a particular subregion. Significance was then assessed by means of a Chi-squared test (*p* < 0.05 corrected for multiple comparisons using Bonferroni’s method) (Clos et al., 2013).

## Results

### Coactivation-Based Parcellation Result

**FIGURE 1:**
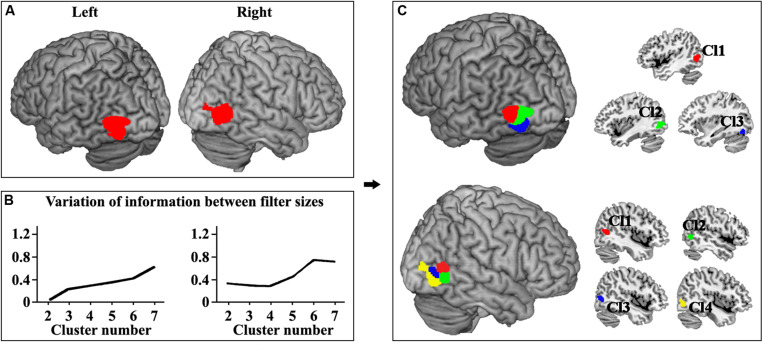
**(A)** The locations of human left and right MT were defined using meta-analysis of visual motion task in BrainMap database. The false discovery rate (FDR) correction with *p* < 0.001 was used. **(B)** The left and right MT areas were parcellated into different clusters ranging from 2 to 7 at the optimal filter size of 115–165 and 100–145, respectively. The hierarchical inconsistency index-identified optimal parcellation schemes for left and right MT were three and four subregions, respectively. **(C)** The three-way parcellation of the left MT (Cl1, Cl2, and Cl3) and four-way parcellation of the right MT (Cl1, Cl2, Cl3, and Cl4) were shown and used to guide the following analyses.

The locations of human left and right MT were defined (Figure 1A). Moreover, the left and right MT areas were parcellated into different clusters ranging from 2 to 7 at the optimal filter size of 115–165 and 100–145, respectively. The hierarchical inconsistency index-identified optimal parcellation schemes for left and right MT were three and four subregions, respectively (Figure 1B). The three- and four-way parcellation of the left and right MT were used to guide the following analyses. In the left MT area, three subregions were identified (Figure 1C). Two subregions were located in the dorsal MT (Cl1 and Cl2), and one subregion was located in the ventral MT (Cl3). The right MT was parcellated into four subregions (Figure 1C). Two subregions were located in the dorsal-anterior MT (Cl1 and Cl2), and one subregion was located in the ventral-anterior MT (Cl3). Moreover, an additional posterior subregion (Cl4) in the right MT was also identified.

### Coactivation Pattern of Each MT Subregion

**FIGURE 2:**
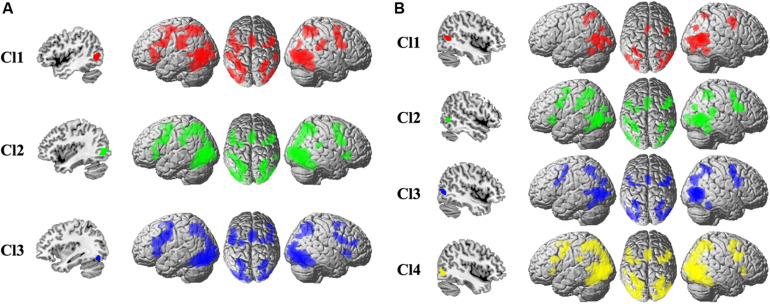
The whole-brain coactivation pattern for each subregion of left **(A)** and right **(B)** MT was obtained using meta-analytical connectivity modeling analyses. The significance levels were set at *p* < 0.05, cluster-level FWE-corrected, and cluster-forming threshold at voxel-level *p* < 0.001.

To uncover the task-related connectivity pattern for each MT subregion, MACM for each subregion was performed, and the coactivation pattern for each subregion was shown in Figure 2. For the left Cl1, the coactivated brain areas were bilateral precentral gyrus (PCG), rostral supramarginal gyrus (SMG), angular gyrus (AG), supplementary motor area (SMA), intraparietal sulcus (IPS), inferior/middle/superior occipital gyrus (I/M/SOG), left inferior frontal gyrus (IFG), and the right posterior superior temporal gyrus (STG). For the left Cl2, the coactivated brain areas were found in bilateral PCG, SMA, AG, IPS, I/M/SOG, left IFG, rostral SMG, right STG, and IFG. The left Cl3 primarily coactivated with bilateral PCG, IFG, SMA, AG, IPS, I/M/SOG, and right middle frontal gyrus (MFG). For the right Cl1, the coactivated brain areas were bilateral IPS, AG, I/M/SOG, SMA, and right PCG. The right Cl2 mainly coactivated with bilateral PCG, SMA, rostral SMG, IPS, I/M/SOG, right STG, and left IFG. The right Cl3 coactivated with bilateral PCG, IPS, AG, SMA, and I/M/SOG. For the right Cl4, the main coactivation was observed in bilateral PCG, IFG, SMA, IPS, AG, and I/M/SOG.

### Specific Coactivation Pattern of Each MT Subregion

**FIGURE 3:**
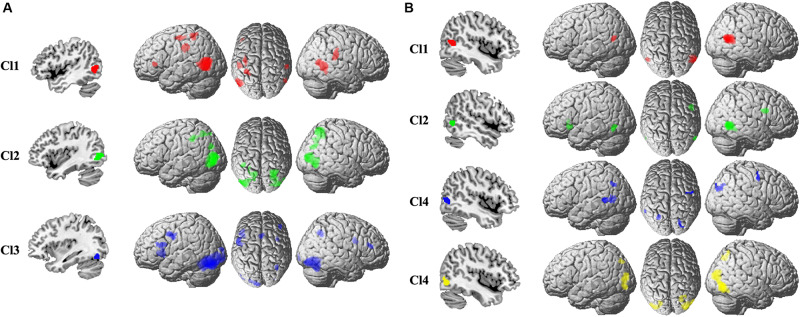
The specific coactivation pattern was calculated to identify the unique task-related coactivation patterns for each subregion in the left **(A)** and right **(B)** MT than other subregions.

The specific task-related connectivity for each MT subregion was mapped to identify the unique coactivation pattern. For the left Cl1, the specific coactivation was found in left IFG, IPS, rostral SMG, right SMG, and posterior MTG (Figure 3). For the left Cl2, the unique task-dependent connectivity was observed in bilateral IPS, AG, and right posterior MTG (Figure 3). The left Cl3 specifically coactivated with bilateral PCG, SMA, IFG, and right SMG (Figure 3). The right Cl1 specifically coactivated with left posterior MTG (Figure 3). For the right Cl2, the unique coactivation was found in left IFG, MT, and right PCG (Figure 3). The specific connection under task for right Cl3 was found in left rostral SMG, posterior MTG, and right PCG, AG (Figure 3). The right Cl4 specifically coactivated with left IPS, posterior MTG, and right SMG (Figure 3).

### Functional Characterization

**FIGURE 4:**
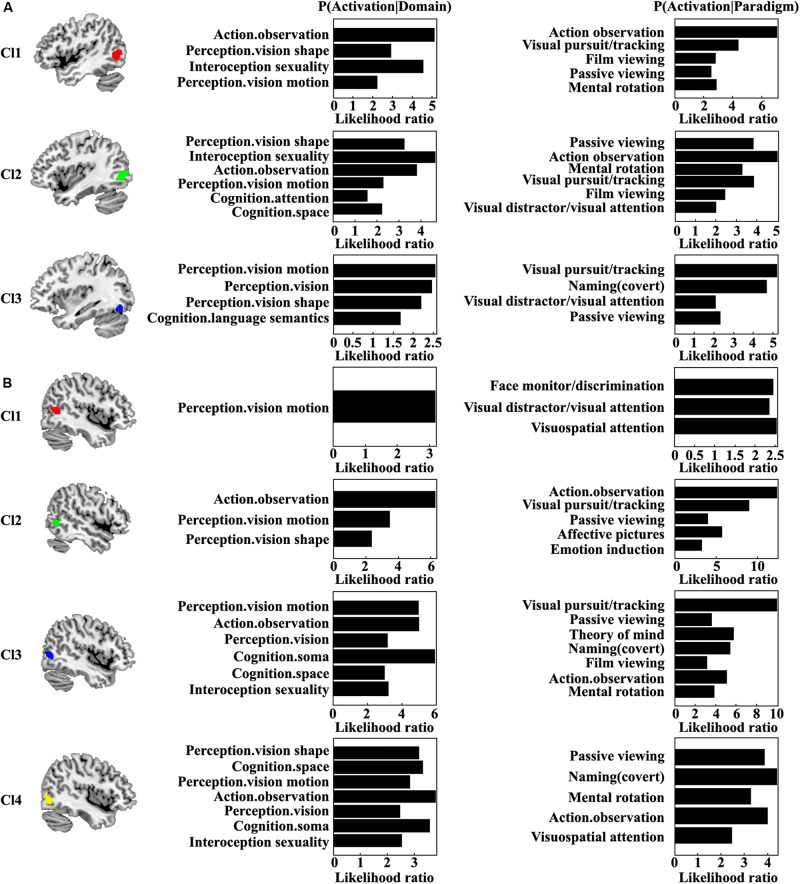
Quantitative functional characterization of each subregion in the left **(A)** and right **(B)** MT was also performed. Significance was established using a binomial test (*p* < 0.05 corrected for multiple comparisons using the FDR method) in the forward inference, whereas it was assessed by means of a Chi-squared test (*p* < 0.05 corrected for multiple comparisons using Bonferroni’s method) in the reverse inference approach.

Quantitative functional characterization of each MT subregion was also performed (Figure 4). For the left Cl1, the main functions associated with this area were action observation and sexuality. The Cl1 was also related to visual shape and motion perception. For the left Cl2, the main functions of this area were sexuality, action observation, and visual shape perception. The left Cl2 was also associated with visual motion perception, space cognition, and attention. The left Cl3 was mainly associated with visual motion and shape perception and semantic processing. For the right Cl1, the main function was visual motion perception. The main function for the right Cl2 was action observation. The functions of visual motion and shape perception for the right Cl2 were also found. The right Cl3 mainly participated in soma, visual motion, and action observation. The functions of visual perception, spatial cognition, and sexuality for the right Cl3 were also found. The right Cl4 primarily participated in activation observation, soma, spatial cognition, visual shape and motion perception, and sexuality. From the coactivation pattern and functional characterization for each subregion, we found that the left subregion of Cl1 (red one) mainly corresponds to the right Cl2 subregion (green one); the left subregion of Cl2 (green one) mainly corresponds to the right Cl3 subregion (blue one); and the left subregion of Cl3 (blue one) mainly resembles the right Cl4 subregion (yellow one). Furthermore, we identified a specific subregion of right Cl1 that is mainly responsible for visuospatial attention processing.

## Discussion

In this study, we proposed a parcellation scheme for the bilateral MT based on whole-brain MACM in the BrainMap database. Three distinct subregions of left MT and four distinct subregions of right MT were identified. In addition to vision motion, distinct and specific functions were identified in different subregions characterized by task-dependent functional connectivity mapping and forward/reverse inference on associated functions.

Notably, multi-functional characteristics of human MT were put forward, supporting that it is not a single area but a complex of areas with several distinct functional subareas. Some of these subareas are likely to be the homologues of monkey MT and its satellites MST and FST (Ferri et al., 2013). To date, many methods were widely performed to reveal the structural and functional definitions of human MT *in vivo*, including motion localizer test (Zeki et al., 1991; Tootell et al., 1995), employing patterns of myelination (Glasser and van Essen, 2011; Bridge et al., 2014), retinotopic mapping techniques (Huk et al., 2002; Kolster et al., 2010), and quantitative T1 mapping (Sereno et al., 2013). The MT was first subdivided into two distinct areas in the monkey brain, one is MT and the other is MST (Huk et al., 2002). Based on more exact measurement of retinotopic map of MT, previous studies have demonstrated at least two retinotopically mapped regions including TO1 and TO2, which correspond to the MT and MST in the monkey brain, respectively (Amano et al., 2009; Kolster et al., 2010). Subsequently, Kolster et al. (2010) segment the motion-sensitive MT cluster into four subregions, respectively, referred to as MT proper, putative MSTv (putative ventral part of the medial superior temporal area), putative FST (fundus of the superior temporal area), and putative V4t (transitional zone) by combining functional MRI and the retinotopic mapping technique. This retinotopic organization in humans was very similar to that documented in the monkey. Shortly after, Glasser and van Essen (2011) used an automated approach to delineate cortical areas based on myelin gradients computed from the ratio of T1- and T2-weighted images collected at 3 T *in vivo*. They identified a large area of strong myelination as the MT complex comprising a number of constituent areas including putative hoc5. However, two previous studies (Abdollahi et al., 2014; Bridge et al., 2014) have compared the definition results of human MT employing patterns of myelination and retinotopic mapping techniques using two complementary approaches. They found that the total number of myelin content within the cortical ribbon was significantly increased. Moreover, Sereno et al. (2013) found that the region of dense myelination on the lateral occipital surface was considerably larger than retinotopically defined MT by comparing quantitative T1 mapping with the retinotopic mapping technique. These results strongly suggested that different studies using different methods resulted in different segmentation of MT regions. Moreover, more challenging is on the way to investigate human myelo-architecture patterns *in vivo* in the human extrastriate regions for individual subjects (Sanchez-Panchuelo et al., 2012). Given these discrepancies and challenging, complementary methods just as we used may provide more additional distinction to reveal the structural and functional definition and parcellation of human MT. Indeed, using meta-analysis of visual motion task in BrainMap database, the locations of human bilateral MT were defined, and they were further subdivided into three distinct subregions in the left MT and four distinct subregions in the right MT based on coactivation-based parcellation in the current study. These results not only were in accordance with the previous findings of a hemispheric asymmetry of MT (Ohlendorf et al., 2008; Kolster et al., 2010), but also strongly confirmed the existence of subregions in this region (Kolster et al., 2010; Bridge et al., 2014).

As expected, all subregions of MT were mainly associated with vision motion. This result was consistent with previous studies that MT is the most known visual MT to detect and signal the presence and direction of visual motion (Lagae et al., 1994; Zeki, 2015). Moreover, most physiological studies showed that many of its cells rather than with that of its component parts are especially associated with the overall, global, direction of an object (Rust et al., 2006), strongly providing the structural fundament of its role in visual motion. Generally, the MT can not only receive information regarding visual motion from the extrastriate area, V6 along a dorsolateral visual stream, but also receive a direct input from V1 and the extrastriate areas of the occipital pole (Zeki et al., 1991). Moreover, evidence showed that neurons of MT are highly sensitive to the speed and direction of visual stimuli in motion (Albright, 1984). It has also been confirmed by several EEG and magnetoencephalographic (MEG) studies (Kawakami et al., 2002; Maruyama et al., 2002; Heinrich, 2007), which showed strong correlations between the latency and amplitude of the evoked response in the visual MT cortical area and the speed of the moving visual stimuli in the adults. Recently, a MEG study showed that the horizontal movement of the visual stimulus evoked changes in the strength of the theta-alpha (5–10 Hz) and alpha-beta (8–20 Hz) oscillations in the visual MT area of all participants (VerMaas et al., 2019).

In addition to visual motion, we also identified distinct and specific functions associated with different subregions characterized by task-dependent functional connectivity mapping and forward/reverse inference. Among these, two findings draw our attention and worth to be emphasized: First, the left Cl2 was also involved in cognition attention based on the results of our functional characterization. Attentional mechanisms also form part of the repertoire of every visual area (VerMaas et al., 2019) but may be utilized differently in different visual areas (Maunsell and Cook, 2002). Several studies showed that the activity of MT can be affected by attention to visual motion (Buchel et al., 1998), because individuals usually attend to the task in the active state. In particular, studies reported modulation of responses in MT from parietal regions involved in selective attention and proposed that these regions modulate the effective connectivity from early visual cortex to the motion-sensitive area MT (Friston and Buchel, 2000). Moreover, our results also showed unique task-dependent connectivity between left Cl2 and bilateral IPS, which is the principal node in dorsal fronto-parietal attention network (Mayer et al., 2006; Connolly et al., 2016). In addition, greater attention-related activations in MT were identified in a task of following arrow cues (Callejas et al., 2014). Considering these information, the previously identified MT associated with dorsal fronto-parietal attention network might be confined to the left Cl2. Second, we also found that the left Cl3 is associated with language semantics. This result was functionally supported by the specific task-dependent connections between left Cl3 and classical language regions, such as IFG and SMG. These two regions have been widely and consistently reported to be associated with semantic processing (Rodd et al., 2015; Xu et al., 2016). Indeed, an fMRI study found that MT was activated significantly more for motion sentences than the other sentence types, suggesting that the neural substrates of linguistic semantics include early visual areas specifically related to the represented semantics (Saygin et al., 2010). Moreover, several behavioral studies also suggested that language can be interacted with low-level motion processing (Meteyard et al., 2007, 2008). However, more evidence is still needed to confirm the role of left Cl3 in semantic processing using more direct approaches.

## Conclusion

In conclusion, we identified two dorsal subregions (Cl1 and Cl2) and one ventral subregion (Cl3) of left MT, as well as two dorsal-anterior subregions (Cl1 and Cl2), one ventral-anterior subregion (Cl3), and an additional posterior subregion (Cl4) of right MT using coactivation-based parcellation. These subregions showed distinct and specific functions characterized by task-dependent functional connectivity mapping and forward/reverse inference in addition to vision motion. These results not only were in accordance with the previous findings of a hemispheric asymmetry of MT, but also strongly confirmed the existence of subregions in this region with distinct and specific functions. Furthermore, our results extend the special role of visual motion perception on this area and might facilitate future cognitive study. Although the MT area was parcellated into different functional subregions using coactivation-based parcellation, future studies with anatomical or resting-state functional connectivity-based parcellation are needed to further validate the current findings.

## Data Availability Statement

Publicly available datasets were analyzed in this study. This data can be found here: www.brainmap.org.

## Author Contributions

All authors listed have made a substantial, direct and intellectual contribution to the work, and approved it for publication.

## Conflict of Interest

The authors declare that the research was conducted in the absence of any commercial or financial relationships that could be construed as a potential conflict of interest.

## Footnotes

1 http://www.brainmap.org/
